# Identification of Risk Factors for African Swine Fever: A Systematic Review

**DOI:** 10.3390/v14102107

**Published:** 2022-09-23

**Authors:** Hannes Bergmann, Johanna Dups-Bergmann, Katja Schulz, Carolina Probst, Laura Zani, Melina Fischer, Jörn Gethmann, Nicolai Denzin, Sandra Blome, Franz J. Conraths, Carola Sauter-Louis

**Affiliations:** 1 Friedrich-Loeffler-Institut, Federal Research Institute for Animal Health, Institute of Epidemiology, Südufer 10, 17493 Greifswald-Insel Riems, Germany; 2 Friedrich-Loeffler-Institut, Federal Research Institute for Animal Health, Institute of International Animal Health/One Health, Südufer 10, 17493 Greifswald-Insel Riems, Germany; 3 Friedrich-Loeffler-Institut, Federal Research Institute for Animal Health, Institute of Diagnostic Virology, Südufer 10, 17493 Greifswald-Insel Riems, Germany

**Keywords:** African swine fever, swine diseases, systematic review, risk factor, epidemiologic factor, Population-Exposure-Comparator-Outcome (PECO), biosecurity, wild boar, Rapid Automatic Keyword Extraction (RAKE), co-occurrence network

## Abstract

African swine fever (ASF) is an internationally-spreading viral pig disease that severely damages agricultural pork production and trade economy as well as social welfare in disease-affected regions. A comprehensive understanding of ASF risk factors is imperative for efficient disease control. As the absence of effective ASF vaccines limits disease management options, the identification and minimisation of ASF-associated risk factors is critical to preventing ASF outbreaks. Here, we compile currently known potential ASF risk factors identified through a systematic literature review. We found 154 observation-based and 1239 potential ASF risk factors, which we were able to group into the following defined risk categories: ‘ASF-virus’, ‘Biosecurity’, ‘Disease control’, ‘Environment’, ‘Husbandry’, ‘Movement’, ‘Network’, ‘Pig’, ‘Society’ and ‘Surveillance’. Throughout the epidemiological history of ASF there have been similar risk categories, such as ‘Environment’-related risk factors, predominantly reported in the literature irrespective of the ASF situation at the time. While ASF risk factor reporting has markedly increased since 2010, the majority of identified risk factors overall have referred to domestic pigs. The reporting of risk factors for ASF in wild boar mostly commenced from 2016 onwards. The compendium of ASF risk factors presented herein defines our current knowledge of ASF risk factors, and critically informs ASF-related problem solving.

## 1. Introduction

African swine fever (ASF) is a transboundary viral pig disease with a massive socio-economic impact worldwide [1]. The causative agent is the ASF virus (ASFV), and infection induces a fulminant haemorrhagic fever with a high case/fatality ratio in domestic and most wild pigs, including European wild boar [2,3]. However, native African suids (particularly warthogs) remain largely asymptomatic following infection [3]. First described in 1921 [4], the disease has remained a worldwide threat to the pig industry to this day [1].

Epidemiologically, ASF is a complex and poorly understood disease. Multiple transmission cycles have been proposed in an effort to describe the role of disease components and potential risk factors for spread within specific socioeconomic and geographical environments [5,6]. In brief, ASFV naturally occurs in the warthog, which represents its main host reservoir on the African continent. The virus is transmitted from the blood of viraemic pigs by a soft tick vector (*Ornithodorus* spp.) that co-habits the burrows of warthogs. Transmission to domestic pigs may occur through contact with infected warthogs, feeding on warthog meat, or by the bite of an infected soft tick vector. For transmission independent of direct contact, it is thought that meat products from infected domestic pigs mostly spread the disease to other pigs [7]. ASFV has high tenacity in the environment and can be recovered from contaminated pig tissues for several months, especially in low-temperature situations [8,9,10,11]. ASFV transmission among European wild boar, likely through ASFV-contaminated wild boar carcass material and direct contact [10,12,13], has formed an unprecedented wild boar habitat-based environmental component to ASFV epidemiology in Eurasia [14,15].

Movement of ASFV-contaminated pig meat is thought to have first disseminated the virus beyond its previous African distribution range to Europe in 1957, then to the Caribbean and South America in 1978 [7,16,17]. Following the first ASFV introduction to Europe in 1957 the virus spread during several infection waves [18], mostly among domestic pig holdings, and was occasionally transmitting to wild boar and feral pigs as well [19,20,21]. Its spread in Europe after 2007 was characterised by two distinct yet seemingly interconnected transmission cycles. Eventually, ASF established a self-sustaining infection cycle in wild boar in Eastern and Central Europe [22]. Following the initial incursion of ASFV into Georgia in 2007 [23], the European ASF arena has further expanded despite concerted disease control efforts [24,25]. From Georgia, ASF spread north in wild boar and domestic pigs, then further west and east, entering EU member states (Poland, Lithuania, Latvia and Estonia) by 2014, and was further spread to several European countries in the following years [22]. In 2018 ASF was first detected in China, from where it spread to a number of Asian countries, thus reaching its largest known range of uncontained spread throughout Eurasia [24,26].

Many risk factors are thought to determine ASFV transmission and regional spread among wild suids or domestic pigs depending on the underlying epidemiological situation [6,27,28,29,30,31]. Thus far, ASF risk factors have been examined with a focus on specific ASF scenarios, including smallholder value chain transmission [32,33,34,35], domestic pig farm transmission [36,37,38,39], and wild boar transmission [37,40,41,42]. However, it is unknown, which types and how many risk factors for ASFV infection in pigs have been observed or proposed, or how risk factors from specific ASF scenarios may interact together to shape the dynamics of disease transmission overall. It is also unclear why ASF continues to spread uncontrollably across diverse ranges of environmental, socio-economic, and jurisdictional settings, highlighting the dependence of ASF spread on as yet unknown or uncontrolled underlying disease risk factors [24]. In order to focus research efforts on addressing verified knowledge gaps [43,44], utilising costly resources efficiently, appraising the risk of disease spread [45], and implementing risk-based disease control measures, a comprehensive knowledge of relevant ASF risk factors is urgently needed.

Here, we systematically searched scientific literature databases to review and identify any potential risk factors considered or observed in relation to ASFV infection in pigs. This work fundamentally supports ASF control by creating a comprehensive portfolio of potential ASF risk factors that have been reported throughout a century of disease history.

## 2. Materials and Methods

### 2.1. Review Protocol and Objective

The methodology outlined herein describes the time-stamped (29 July 2019) systematic review protocol which was developed a priori for the identification of currently known ASF risk factors. The authors have not conducted this review previously. The review protocol was developed according to the PRISMA (Preferred Reporting Items for Systematic Reviews and Meta-Analysis) guidelines as applicable for the study objective (Table S1) [46,47]. The purpose of this study was to identify any potential risk factors considered or observed in relation to ASFV infection in wild or domestic pigs, thus informing the following systematic review objectives:

1. **Primary objective:** Identify and summarise available information on ASF risk factors for which predefined observations have been reported in relation to ASFV infection in domestic or wild pigs (***observation-based risk factors***).
2. **Secondary objective:** Identify and summarise hypothesised risk factors that have been mentioned in the literature for ASFV infection in wild or domestic pigs, but for which observations have not necessarily been made or reported (***potential risk factors***).

### 2.2. Eligibility Criteria

To identify reported ASF risk factors, this review included as many primary peer-reviewed studies about ASF risk factors as were accessible until the search cut-off date. Thus, no time limitations were applied when retrieving search results other than the limits inherently imposed by the coverage timespan of the utilised databases and the search language. Review articles and editorial-type letters were excluded. Consistent with the language cognition accessible to the reviewer team, records that were not available in English, German, Italian, or Spanish were excluded. To identify relevant studies that included observations about risk factors associated with ASFV infection and risk exposure in pigs, the PECO (Population-Exposure-Comparator-Outcome) principle was applied to structure the necessary criteria of risk exposure reports according to the review objective [47,48]. Based on the PECO structure, observation-based eligibility criteria for studies of interest were defined to assess the inclusion of records for review and ASF risk factor identification:

- Population (P): the study describes domestic or wild pigs exposed to ASFV
- Exposure (E): the examined study population is exposed to a defined risk factor
- Comparator (C): the study describes pigs that are exposed to ASFV but not to the examined risk factor, or a suitable reference scenario is described
- Outcome (O): the studied pig population is infected with ASFV as determined by some form of measurement

Identified eligible records for which a full text document was not available by 20 November 2020 were excluded from the review.

### 2.3. Information Sources

To identify literature-based ASF risk factors, eight separate databases were queried. The inherent timespan of database coverage was applicable as assessed through open time-based search queries, for example, using the string “1700:2100(dp)” in PubMed or “PUBYEAR > 1700” in Scopus. The known timespan of database coverage or the oldest record retrieved from open search queries (if older than the known year) and their accessed web-addresses were:

- MEDLINE via PubMed (1946 and selected coverage from 1781, https://pubmed.ncbi.nlm.nih.gov/, accessed on 30 September 2020)
- MEDLINE via Web of Science (1945, https://apps.webofknowledge.com/, accessed on 30 September 2020)
- Scopus (1970 and selected coverage from 1788, https://www.scopus.com/, accessed on 30 September 2020)
- EFSA Journal (2003, https://efsa.onlinelibrary.wiley.com/, accessed on 30 September 2020)
- AGRIS (1965, https://agris.fao.org/agris-search/, accessed on 30 September 2020)
- Open Theses and Dissertation (annotations from 1971, https://oatd.org/, accessed on 30 September 2020)
- Networked Digital Library of Theses and Dissertation (annotations from 1971, http://search.ndltd.org/, accessed on 30 September 2020)
- DART-Europe (1999, http://www.dart-europe.eu/, accessed on 30 September 2020)

These databases were selected in order to capture the primary scientific literature in the fields of biomedical sciences, agriculture, veterinary medicine, social sciences, animal sciences, and economical sciences, all of which were deemed relevant for the review objective. Required full-text records were obtained through professional in-house library services utilising online requests and local repository access.

### 2.4. Search Strategy

Suitable search strings were designed to search for literature records of interest (Text S1). Search string design was supported by professional library services with extensive experience in bibliographic searches. A detailed description of the search strategy is provided in Text S2. Searches were conducted on 21 August 2019 and updated on 30 September 2020. For each search, all retrieved results were recorded and extracted to assemble a search library suitable for the study selection process and subsequent review. The extracted record data were stored and managed with Endnote X9, version number 12062 (Clarivate, Philadelphia, PA, USA) and using the ‘revtools’ package in R software version 3.6.3 (http://www.r-project.org, accessed on 28 March 2022) [49].

### 2.5. Study Selection

**Figure 1:**
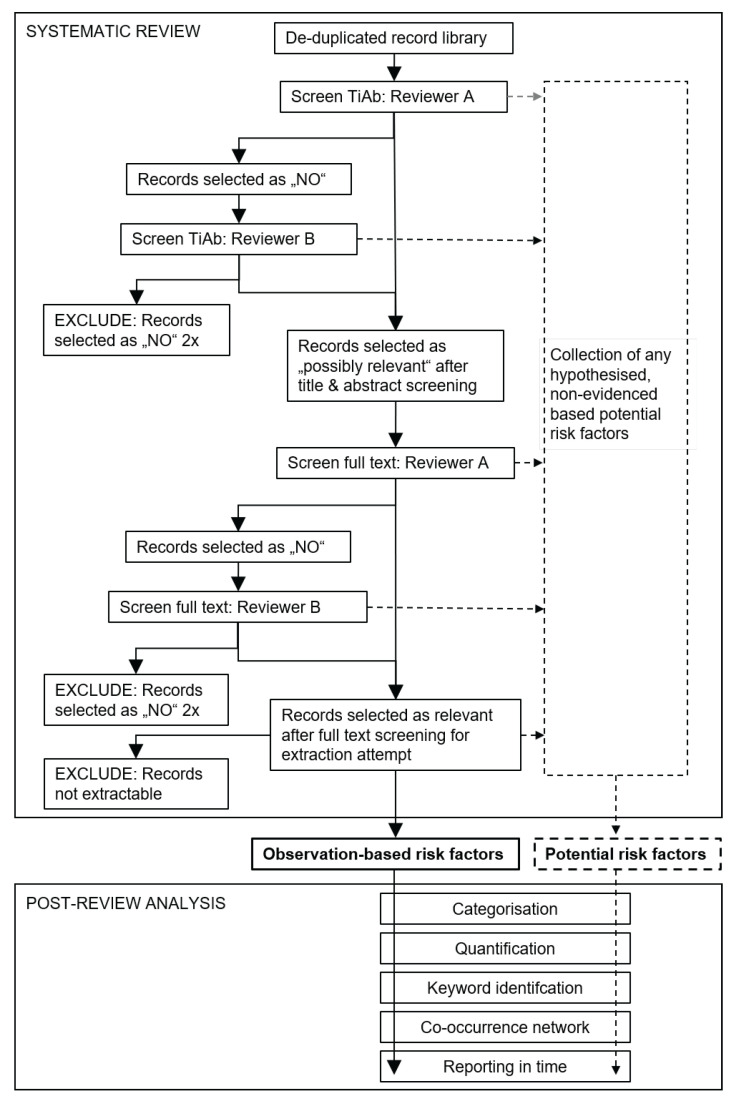
Schematic flow diagram of the literature screening procedure and post-analysis applied to de-duplicated African swine fever search libraries. If a record was deemed ineligible for the review questions, it was assessed again by a second independent reviewer for possible inclusion. Risk factors identified by systematic review were further examined in a post-review analysis. TiAb, title and abstract.

Starting with the compiled search library of retrieved records, studies were selected in a stepwise manner as summarised in Figure 1 and Table 1.

Text S2 describes the selection process in more detail. Comments made by the reviewers as part of the potential risk factor collection process (dashed lines in Figure 1) were collated, conservatively de-duplicated by exact matches from the same record, and categorised for structured presentation.

### 2.6. Data Collection

Eligible studies selected for attempted extraction were evaluated and the ASF risk information was extracted where possible. Extraction was performed by one reviewer, after which a second reviewer independently crosschecked and confirmed the recorded data. Any disagreements were discussed and resolved by reaching consensus. Standardised electronic data extraction forms were used to record study information in an extraction matrix. In line with the review objective, the following data were extracted: Risk factor, Risk factor category, World region of study, Study area, Pig type, Author, Year, Title.

### 2.7. Post-Review Analysis of Risk Factors

Following the identification of risk factors through systematic review, the collected information was further analysed in order to better summarise its complexity. The identified risk factors were categorised and quantified, key words were extracted, relationships between risk categories were examined using co-occurrence word networks, and the reporting of the risk factors over time was inspected (Figure 1).

#### 2.7.1. Categorisation of Risk Factors

In order to comprehend the extracted risk factor information in a meaningful way, a method of grouping and synthesising them was necessary. We categorised the identified risk factors using an epidemiological triad and disease components for ASF [48,50,51]. Risk categories were created based on disease elements relevant for ASF occurrence as indicated by the epidemiological triad and based on the types of categories necessary to describe the identified risk factors in the literature. Consequently, the resulting risk categories were thematically informed by the risk factors found in the literature and organised according to the epidemiological triad. The epidemiological triad typically includes the environment, host, and pathogen as components of disease causation [48,50], although these elements may be expanded and populated with components to suit the disease of interest. Each ASF risk category used to thematically group the identified risk factors was described, and the risk factors were allocated to categories according to these descriptions.

#### 2.7.2. Quantification of Risk Factors by Risk Category

The identified potential and observation-based ASF risk factors were counted and plotted as bar graphs for each assigned risk category. For observation-based risk factors, the examined pig type could be extracted from the literature and thus considered for the enumeration of risk factors in each category. Depending on the literature reports, this meant that the same risk factor may have been counted several times in each risk category; here, risk factors were counted once at most in association with each considered pig type.

#### 2.7.3. Rake Keyword Identification

To determine the keywords that were mentioned most consistently among the detected risk factors within each risk category, a rapid automatic keyword extraction algorithm (RAKE) was applied to the identified ASF risk factor text [52] using the R software package ‘udpipe’ [53]. More detail about this method is provided in Text S2.

#### 2.7.4. Co-Occurrence Word Networks

In order to examine the literature-based representation of individual risk factors in association with the risk categories, as well as to uncover the complexity with which individual ASF risk factors were considered to interact and influence disease, we conducted a co-occurrence network analysis of the risk information. A detailed method description is provided in Text S2. In brief, words were annotated with the Universal Dependencies treebank language model ‘English-partut-ud-2.5-191206’ [54], co-occurrences were calculated with the R software package ‘udpipe’ [53], and network relationships were visualised with the ’ggraph’ package [55].

#### 2.7.5. Temporal Pattern Analysis of Risk Factor Reporting

To discern the distribution of ASF risk factors considered by the scientific community over time, the frequency of identified ASF risk factors and the associated risk categories were examined for all publication years covered by the systematic search results; refer to Text S2 for more detail.

## 3. Results

### 3.1. Observation-Based Risk Factors Found by Systematic Review

**Figure 2:**
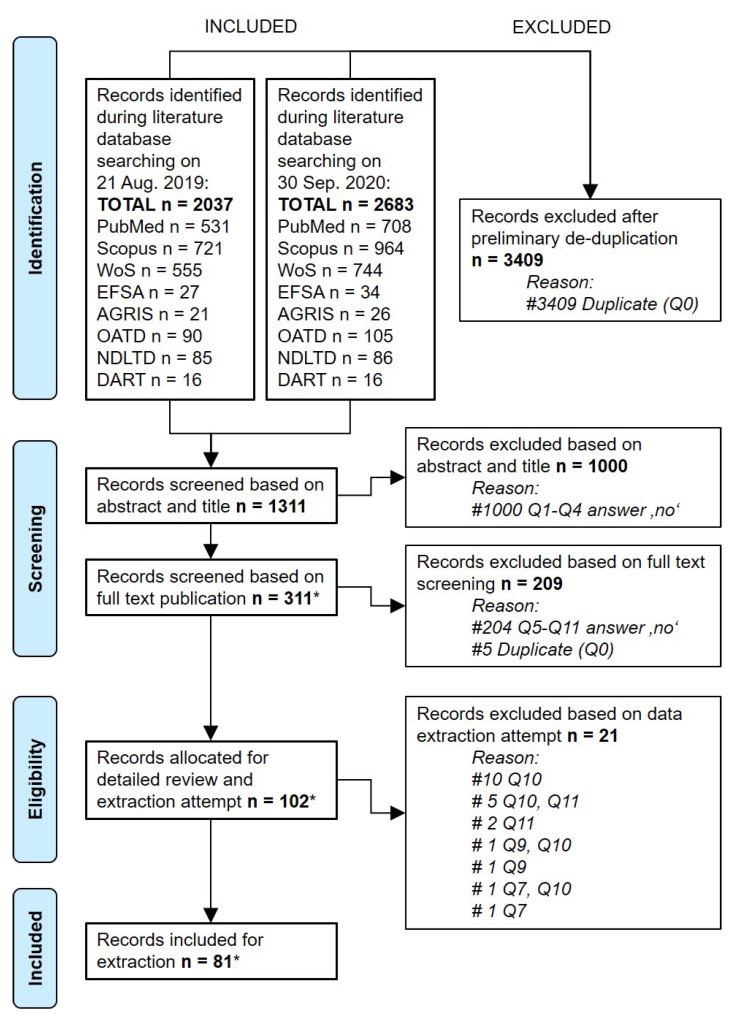
PRISMA flow diagram of included and removed records screened in the search library for African swine fever risk. Q, selection question (see Table 1). * includes non-peer reviewed EFSA reports.

A total of 4720 literature records were retrieved and compiled into a single search library after two subsequent database searches had been conducted, with the initial search on 21 August 2019 yielding 2037 records and the search update on 30 September 2020 producing 2683 records. The library was subsequently subjected to a stepwise selection and data collection process (see Figure 1 and Figure 2 and Table 1).

Altogether, 3409 records were excluded from the library through de-duplication, resulting in 1311 remaining records, which were then submitted to the literature screening procedure by the reviewers (Figure 2). Screening of the records against the PECO-based eligibility criteria led to the exclusion of 1000 records based on their titles and abstracts and the selection of 311 records for full text screening. Review of the available full texts resulted in the exclusion of a further 209 records from the original library (Table 1 and Table S2).

The remaining 102 full texts were considered for extraction of risk factor information. During the extraction attempts, it became evident that 21 records did not yield extractable risk factor information, and these were excluded as well (Table S2). The final library of 81 records provided data for the identification of 154 observation-based risk factors (Figure 2). Details about the selected literature records are provided in Table S3. All identified observation-based ASF risk factors that were examined in the reviewed literature are presented in Table S4.

If the multiplicity of distinct pig types had to be accounted for here, 210 observation-based risk factors would have to be reported, as several risk factors were examined in multiple identified pig types. Moreover, several risk factors were examined by multiple studies, thus further increasing the number of unique risk factors to 411 if the factors were differentiated by literature record (Table S4).

### 3.2. Potential Risk Factors Found by Systematic Review

In addition to the systematic identification of observation-based risk factors described above, and according to the second objective of this work, an exhaustive search for potential ASF risk factors was conducted (Figure 1). The search identified 467 publications that elicited comments by the reviewers during the literature screening, resulting in a collection of 1239 potential risk factors (Table S5).

Many of the collected potential risk factors were found to be mentioned repeatedly or with only slight variations across the examined documents. While identical (duplicate) risk factor notations were removed from the collection, similar or re-worded potential factors were intentionally retained, as they often suggested consideration of different aspects within an element or pathway of potential disease risk. It was deemed more important to capture the widest possible spectrum of previously considered risk factors from the screened literature rather than possibly losing a potentially idea-provoking element.

### 3.3. Categories Applied to Risk Factors

Based on the identified ASF risk factors, ten risk categories were assigned to the observation-based risk factors, namely, ‘ASFV’, ’Biosecurity’, ‘Disease control’, ‘Environment’, ‘Husbandry’, ‘Movement’, ‘Network’, ‘Pig’, ‘Society’, and ‘Surveillance’.

**Figure 3:**
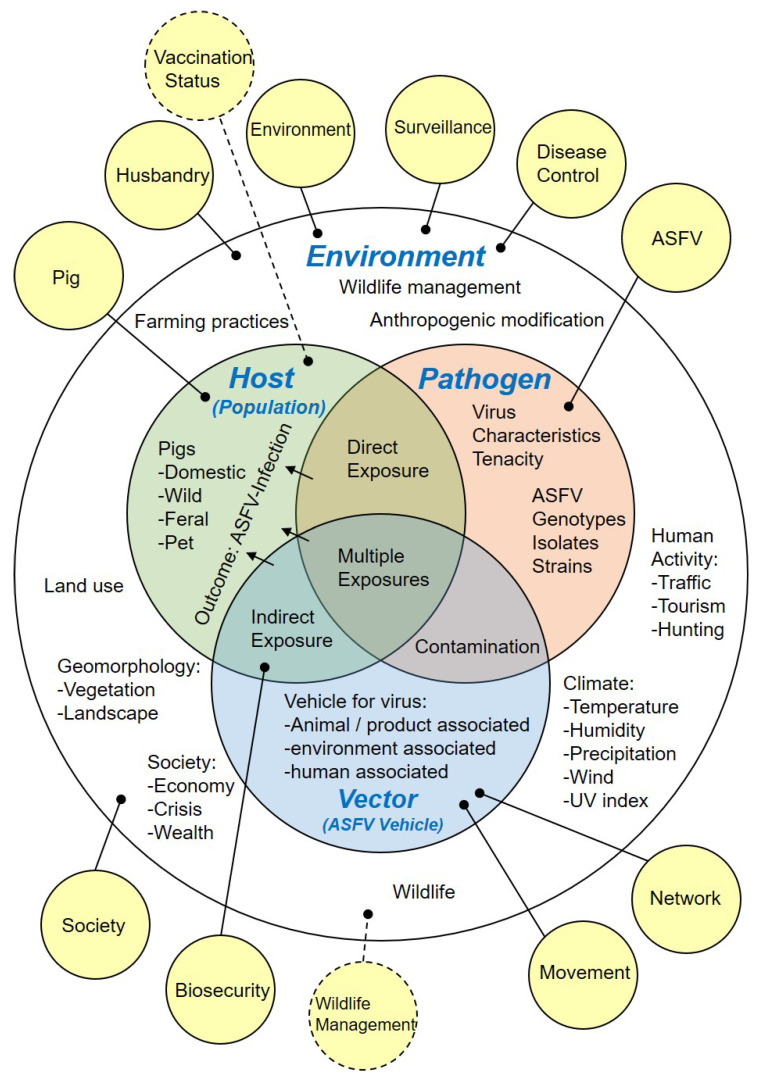
Epidemiological triad modified to structure possible disease determinants relevant for ASF occurrence (Environment, Host, Pathogen, Vector). Solid yellow bubbles indicate observation-based risk categories, while dashed yellow bubbles indicate additional potential risk factor categories. ASFV, African swine fever virus; UV, ultra violet.

In order to thematically group the identified potential risk factors, two additional risk categories were assigned to capture the spectrum of identified information, namely, ‘Vaccination status’ and ‘Wildlife management’. All allocated risk categories are summarised in Figure 3.

A description of each risk category is presented in Table 2.

The allocation of risk categories to observation-based risk factors, including the applicable world region and examined pig type, are summarised in Table S4. Potential risk factors are summarised by category in Table S5.

We introduced risk categories in order to summarise our findings and convey meaning in the context of managing disease determinants for ASF (Table 2).

### 3.4. Quantification of Risk Factors Found

ASF is an epidemiologically complex transboundary disease that has globally evolved over time and across multiple ecosystems to involve different types of pigs and transmission pathways [5]. We therefore considered it important to link the identified risk factors with the epidemiological background. This was achieved by analysing the frequency of the collected risk factor information in a thematic and temporal context.

**Figure 4:**
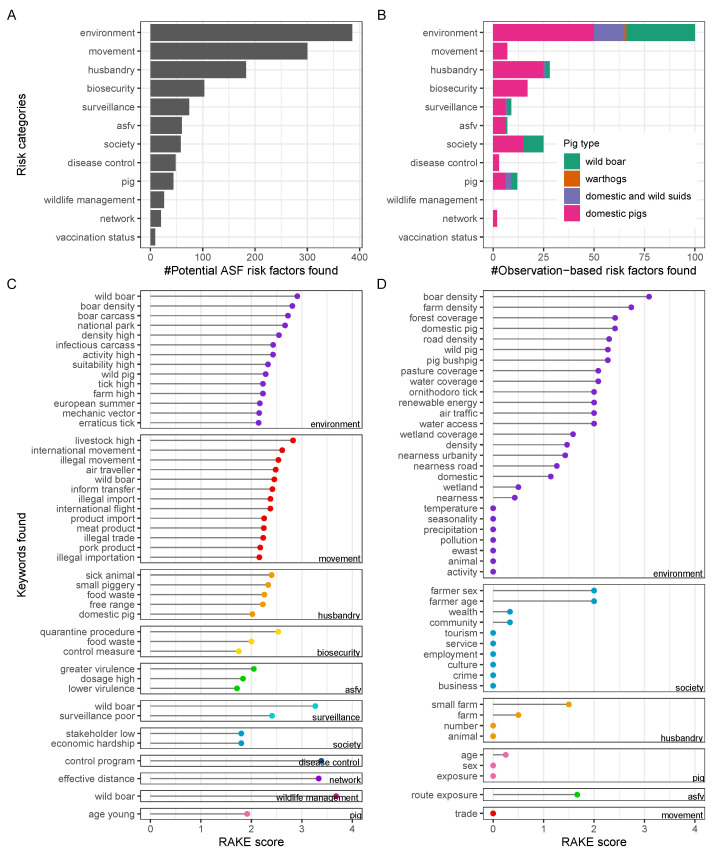
ASF risk factors. Enumeration of potential ASF risk factors (**A**) and observation-based ASF risk factors (**B**) by pig type and category. Keywords identified through RAKE among potential (**C**) and observation-based (**D**) risk factors. RAKE, Rapid Automatic Keyword Extraction Algorithm; ASFV, African swine fever virus.

According to the assigned risk categories (Table 2), ASF risk factors can be thematically distinguished by, for instance, whether they are related to the pathogen, the host, or the environment. It was found that ASF risks related to environmental factors were reported with the highest frequency, making this risk category the most diverse for ASF. As shown in Figure 4A,B, this was found for both potential and observation-based risk factors.

Among potential risk factors, movement, husbandry, and biosecurity-related risks were the next most diverse that were identified here (Figure 4A). This was in contrast to the number of unique observation-based risk factors found related to movement, which was much lower relative to the other risk categories (Figure 4B). In addition, the relative number of unique observation-based risk factors in society and pig-related ASF risks was higher. These findings indicate that the level of attention certain potential ASF risks have received in the scientific literature is not reflected by the available reporting investigating related observations in ASFV-infected pigs.

Regarding pig type, identified observation-based risk factors were differentiated as to whether they related to domestic pigs and/or wild suids such as warthogs or wild boar (Figure 4B). We found that wild suid-related observation-based risk factors were mainly included in the risk categories with the highest detected frequency, such as environment, society, and husbandry. Noteworthy risk categories that did not relate to wild suids, only exclusively to domestic pigs, included ‘biosecurity’, ‘movement’, and ‘disease control’.

### 3.5. Keyword Risk Terms Identified

Keywords extracted from the identified ASF risk factors are shown grouped by risk category in Figure 4C,D.

The following keywords were found among potential risk factors (Figure 4C): Those related to environmental factors were wild boar, boar density, infectious carcasses, farms, tick vectors, and season. Those for movement-related factors were movement of livestock, wild boar, pork products, and air travellers through international, informal, or illegal transfer. Those regarding husbandry practices were sick animal management, smallholdings, food waste, or free range. Those for biosecurity risks were quarantine procedures, food waste management, and implementation of control measures. ASFV pathogen-related risk keywords were high viral dosage and both higher and lower ASFV virulence. For the remaining risk categories, poor surveillance, particularly of wild boar, societal hardship, insufficient disease control programs, effective network distances of relevant epidemiological units, wildlife boar management, and younger pig ages were found as keywords (Figure 4C).

Fewer keywords were identified among observation-based ASF risk factors, which is consistent with the lower number of factors found for this type overall. Wild boar or wild pig density, farm density, forest coverage, domestic pigs, road density, pasture coverage, and water or wetland coverage were among the diverse group of keywords related to environmental ASF risk factors with an observational basis (Figure 4D). Farmer’s sex and age as well as wealth and community factors were identified as keywords among society-related risks. Small farm husbandry, pig age, ASFV exposure routes, and movements through trade were detected as keywords of observation-based ASF risk factors in the less diverse categories (Figure 4D).

A summary of all keywords that were found through RAKE among potential and observation-based ASF risk factors is provided in Table S6.

### 3.6. Risk Term Co-Occurrence Links between Categories

**Figure 5:**
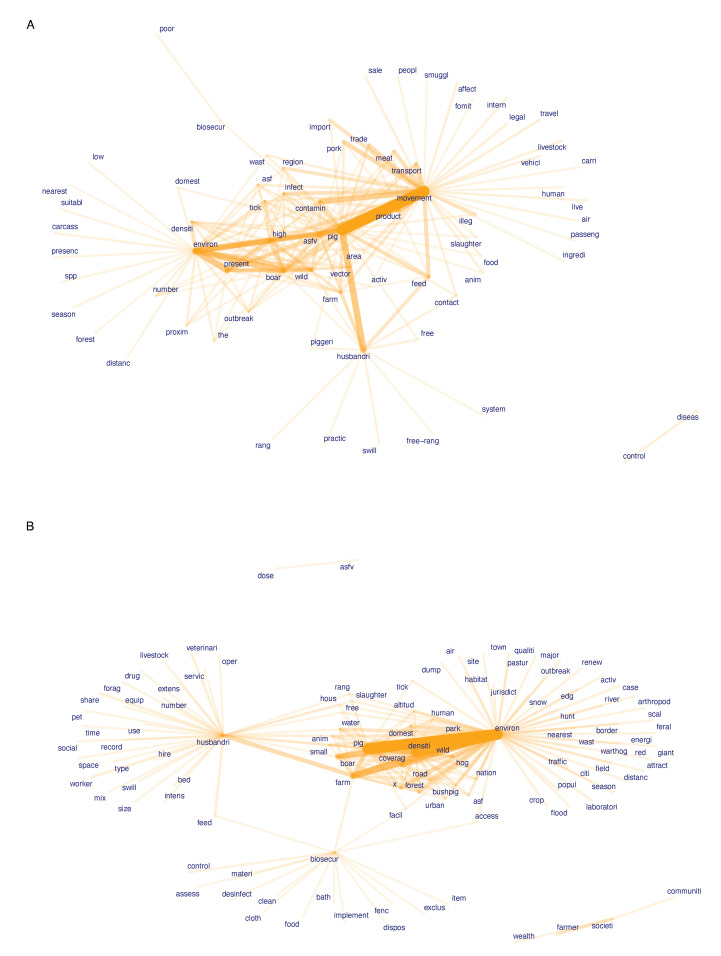
Co-occurrence networks of keyword stems among identified potential (**A**) and observation-based (**B**) ASF risk factors. The top 200 co-occurring words are shown. Orange bar thickness represents the frequency of co-occurrence.

We used word co-occurrence network graphs to identify clustering and linkages among the examined ASF risk factors and risk categories (Figure 5A,B).

Among potential risk factors the environment, movement, and husbandry categories formed the main clusters, mainly reflecting the large number of diverse factors identified for these risk categories (Figure 5A). ‘Farm’ and ‘boar’ were found as co-occurring links between environmental and husbandry-related risk keywords. ‘Feed’ and ‘contact’ words linked husbandry with the movement cluster, whereas ‘contamination’, ‘infection’, ‘region’, and ‘waste’ were among the linking words for movement and environmental risks. ‘Pig’ was a central term in the network, indicating common (though likely unspecific) usage among all risk categories. Although biosecurity-related potential risk factors were identified as the fourth most frequent category (Figure 4A), biosecurity was not well linked with other co-occurrence clusters (Figure 5A), indicating that this category described a distinct set of potential ASF risks.

By contrast, the biosecurity word cluster appeared more interlinked when observation-based risk factors were examined in a co-occurrence network (Figure 5B). In addition to biosecurity, husbandry-related and environment-related factors formed the main interlinked word clusters. Biosecurity was linked with the husbandry cluster through ‘feed’ and ‘farm’ and with the environment cluster through ‘facility’ and ‘access’. The environment cluster was linked to multiple husbandry related words, including ‘farm’, ‘boar’, ‘small’, ‘water’, ‘free’, ‘range’, ‘housing’, and ‘slaughter’. Other central words in the network were ‘density’ and ‘pig’, highlighting their common usage. Words from the society and ASFV risk categories formed isolated clusters, despite a larger number of risk factors that were found for the society category (Figure 4B). This indicates that the literature has examined a distinct set of risks, particularly in this category.

### 3.7. The Temporal Pattern of Risk Factor Reporting

**Figure 6:**
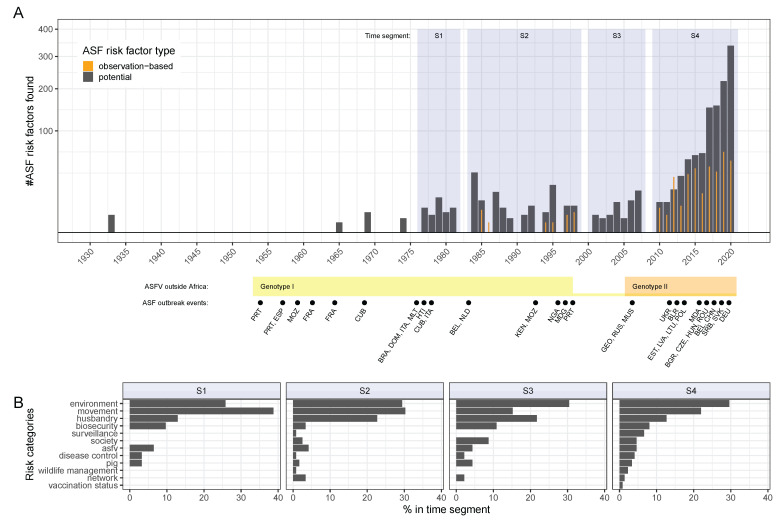
Timeline of potential and observation-based ASF risk factors reported in the literature in relation to time segments of ASF events in the world (**A**). Only ASF genotypes detected outside of Africa are indicated along the time axis. Country names are abbreviated by their ISO 3166 ALPHA-3 code. Categorical distribution of literature-reported potential ASF risk factors in sequential time segments (**B**). Time segments are: S1: 1977–1981, S2: 1984–1998, S3: 2001–2007, S4: 2010–2021. ASFV, African swine fever virus.

The chronology of risk factor reporting in the literature was examined. The earliest record retrieved by the systematic searches addressing potential ASF risks was published in 1933 [56]. No risk factor information was found from 1933 through the 1960s. Until 1985, only potential risk factor information was retrieved, with no observation-based risk factor studies selected prior to this time (Figure 6A).

Of the observation-based risk factor studies, 9 of 81 (11%) were published between 1985 and 2010. All remaining 72 studies were published from 2010 onwards (Figure 6A and Table S3). Identified potential risk factors showed a similar profile, with the availability of ASF risk information starkly increasing from 2010 onwards.

Key historic ASF events have shaped the epidemiological disease situation in the world over time (Figure 6A, lower panel) [17,18,57,58,59]. Accordingly, it could be expected that ASF risk factors of interest would have changed over time as well and that this would be reflected in the scientific literature on the type of risk factors reported. It was found, however, that this was not the case (Figure 6B). The retrieved risk information was organised into four time segments for the years of 1977 to 1981, 1984 to 1998, 2001 to 2007, and finally 2010 to 2021. When comparing the proportion of potential ASF risk factors reported for each risk category during these time segments (S1 to S4), we found that the types and frequency remained relatively constant over time (Figure 6B). The most frequently identified risk categories across all time segments and in descending order were environment, movement, and husbandry. Although, the proportion of risk factors related to movement were considered more frequently than environmental factors during the earlier time segments between 1977 and 1998.

The selected literature records reporting observation-based risk factors were differentiated based on the examined pig types (Tables S3 and S4). The majority of records, 56 of 81 (about 69%), were found to address risk factors only in domestic pigs, while 18 records examined risks in wild boar (22%) and 6 records looked at domestic pigs and wild suids (7%). Only a single record investigated risk factors in warthogs. With the exception of this one warthog related study in 1985 and an ASF examination of European wild boar in 1998 [20,60], all selected records reporting observation-based risk factors for ASF in wild suids, including European wild boar, were published in 2016 or later (Table S3).

## 4. Discussion

Although ASF has now been managed for over 100 years [4], stopping its spread appears to be nearly impossible, as highlighted by the current epidemics traversing Europe and Asia [18,24,61]. To halt ASF spread, there is an urgent need to understand what makes it so difficult to control the disease. It is possible that we are either missing awareness of critical risk factors for transmission or that we are unable to implement effective control measures despite sufficient risk factor knowledge.

Here, we provide a basis for addressing this fundamental problem by creating a library of the currently available scientific information about ASF risk factors through a systematic literature review. We employed two separate approaches in parallel to identify potential and observation-based ASF risk factors in the reviewed scientific literature databases (Figure 1 and Table 1). To comprehend the identified ASF risk information, we structured it through categorisation (Figure 3 and Table 2) and analysed the frequency with which risk factors were mentioned in the reviewed literature by time and risk category (Figure 4).

Furthermore, we used a keyword extraction algorithm and word co-occurrence networks to provide additional information by identifying relevant risk terms and showing interconnectedness among the identified risk factors (Figure 5). While it is beyond the scope of this work, further analysis of these types of data are necessary to reveal the links within the available risk information, possibly by examining temporal, author, species, and spatial relationships among the identified studies.

Taken together, this systematic, literature-based review summarises the status quo of reported ASF risk factors to date. This knowledge is fundamental to understanding whether important risk factors remain to be discovered and where the efficacy of current ASF control measures could be improved.

More detailed analysis of the identified risk factors granted complementary insights. By analysing the frequency and categorical type of identified risk information over time, we examined whether ASF risk factors are associated with the concurrently occurring ASF scenarios around the world (Figure 6) [17,18,57,58,59]. We hypothesised that the quantity and diversity of ASF risk information would increase during or shortly after outbreak events. We further hypothesised that the prevailing outbreak arena and specific epidemiological context would influence the type of risk factors studied. It appears plausible that ASF-induced crises necessitate contemporaneous interest and funding to investigate disease mechanisms [17,44].

In addition, it has been postulated that interlinking transmission cycles epidemiologically define ASF spread mechanisms and associated risk factors depending on the dominant disease arena [6]. As it thus appears necessary to provide disease scenario context in order to understand the identified ASF risk information, it might therefore be expected that the risk factor types examined here would reflect the prevailing ASF scenario and associated transmission cycle. Surprisingly, we found that the opposite was the case. By grouping risk information into distinct annual periods and extracting the risk categories most frequently identified during each time segment, we found that there was little variation in the main types of ASF risk subjected to scientific examination throughout the entire history of ASF, regardless of temporally associated outbreak scenarios. However, in line with our expectations, the quantity of identified risk information appeared partially associated with the global ASF situation, in that increased risk information was reported during outbreaks.

This was particularly true for the currently ongoing Eurasian ASF epidemic, which started in 2007 in Georgia [23]. The most frequently identified risk categories for potential risk factors throughout ASF history were environment, movement, and husbandry. This shows that the environment category is overrepresented, and that this has happened consistently over time. This finding is in line with the fact that ASF has been described as an environmental disease [15,62,63].

We wondered why the same risk categories were most frequently identified regardless of the timeframe. It is possible that the same risk categories were deemed relevant for reporting on for all ASF scenarios encountered over time regardless of the transmission cycles involved. Alternatively, the distinction of categorical risks among different ASF transmission cycles and outbreak arenas may be far less pronounced than is generally assumed [6,64]. Moreover, scientific examination of seemingly distinct outbreak scenarios ultimately may have led to comparable risk categories. The latter explanation is consistent with the observation that movement was not included in frequently identified risk categories through systematic selection, which revealed ’pig’ as a frequently reported risk category instead.

Simply put, the potential risk factors collected here most likely represent ideas that could be considered as risk factors for ASF. Observation-based risk factors found through systematic selection, however, represent matters for which observations in ASFV-infected pigs have already been reported.

This perspective implies that while movement as potential risk has been a commonly considered factor for ASF spread for many years (Figure 6) [7], the number of associated observations identified in ASFV-infected pigs is low (Figure 4A,B). This indicates that prevailing assumptions about ASF risks do not necessarily have an observable basis in the literature, even though movement restrictions and zoning are fundamental to animal disease control. A likely explanation for this observation lies in the difficulty of obtaining suitable data for examining movement-related ASF risks.

One conclusion that can be drawn from the identified lack of variability among frequently identified risks over time is the need to encourage researchers to stray off the beaten path and help reveal previously unobserved connections between potential risk factors and ASFV infection in pigs. To expand the conventional ASF research focus beyond environment and husbandry risk categories, the list of potential ASF risk factors purposefully assembled here (see Table S5) may provide a helpful starting point. In this sense, the categorical frequency analysis of identified risk factors could help to identify and prioritise readily translatable ASF research gaps or highlight areas for which risk information is scarce [44,65].

Categorical enumeration of the identified risk factors highlights that certain risk categories are relatively underrepresented, indicating that factors contained therein have not yet been observed to influence the risk of ASF spread as perhaps expected. ASFV is one of these categories, indicating that pathogen-related factors such as genetic variance potentially provide a much smaller than anticipated basis of observation for informing ASF strain-related risks through molecular tracking of pathogen characteristics and epidemiology [66].

Our systematic literature review revealed that the types of risk factors identified throughout ASF history are categorically similar regardless of the concurrent ASF situation at the time, that risk factor reporting has particularly increased since 2010, and that there is a focus on environmental risk factors in the ASF risk literature, mostly in domestic pigs.

As in any literature-based work, the present study has limitations. This systematic review focused on the identification of potential ASF risk factors. Thus, a wide search strategy was combined with selection criteria-based screening and review of records to find as many previously considered risk factors as possible. Of the identified risk factors and studies, we deemed it more important to include a wide range of risk information than to impose stringent restrictions through strict application of quality measures in this context. Specifically, we observed variations in the fulfilment of selection criteria for the PECO-informed selection of records reporting ASFV infection in pigs with exposure to risk factors. The risk of bias on the study level includes variation in study type or the epidemiological context referred to, for example, the country of study. This information may be important for the evaluation of risk factor evidence, which was beyond the scope of this work.

Risk of bias in the selection of articles for our library may have arisen from applying selection criteria at initial screening that were too narrow or limiting, such as language specification and exclusion of reviews (Table 1). We used only English for the search terms. The search focused on terms related to ‘risk’ and ‘African swine fever’, which may have limited identified records to publications stating these terms in the English language. Thus, other potentially relevant bodies of work in non-English languages or with historically alternative descriptors for ‘risk’ and ‘African swine fever’ may have been ignored.

The choice of queried databases and subjective collection of potential risk factor types by the reviewing team could have led to incomplete or non-representative identification of ASF risk information. While these limitations have to be considered in the interpretation of our findings, we judge the overall collection of ASF risk factors in this review to be near complete. This view is based on the following two observations. First, while many of the collected potential ASF risk factors turned out to be similar, these were retained in the final library and many of the originally extracted factors were removed as exact duplicates, indicating saturation of risk factor identification. Second, search string queries of the chosen literature databases initially retrieved 2037 records, yet de-duplication then excluded almost half of these records (Figure 2). This suggests that the coverage of our search was almost completely redundant among the targeted databases. The possibility of missing reported ASF risk factors or previously considered risk factor ideas within the search frame and the targeted search terms in this review can therefore be considered low.

Categorisation was applied to ASF risk factors, and the decisions with respect to assigning a risk factor to a specific category were to some degree made subjectively despite implementing descriptions for each category (Table 2) [51,67]. While the categorisation fulfilled its intended purpose of thematically structuring the identified risk information, we did not assume that the applied categories were exclusive or complete. Conclusions based on category allocation decisions should be viewed in relation to the chosen grouping of risk factors, and might shift if the risk factor-category assignment were changed or categories were removed or added.

To exemplify this point, the following cases should be considered. The increased occurrence of diverse ASF risk categories in the literature during distinct time segments is independent of the chosen categorisation due to the influence of data stratification on diversity counts being minimal (Figure 6B). By contrast, examination of risk factor frequency by category is only informative for the chosen set of defined categories (Table 2, Figure 4 and Figure 6). Moreover, the quantity of factors found for each category is unlikely to be indicative of the category’s true natural relevance for disease causation, and instead should be understood as representing an expert measure of importance in accordance with the collective perspective of the scientific ASF community. As such, frequently identified categories in the literature could be interpreted as worthy of research time and resource investment, highlighting the relevance of the topic [68]. In this way, category-associated frequency measures can be useful for allocating a relative weight to each risk category, and by extension to the risk factors within these categories (Figure 4A,B).

## 5. Conclusions

This systematic review identified a comprehensive collection of known ASF risk factors. Our findings can help to guide the identification of previously unrecognised risk factors, reveal research gaps, support the evaluation of ASF management strategies, and inform risk assessments.

## Figures and Tables

**Table 1:** List of inclusion and exclusion criteria for sequentially applied literature selection steps. Selection questions were consecutively applied to all records during the literature screening procedure. The stages of the screening at which each of the questions were used are indicated, as well as possible answers and the corresponding consequence for the selection process. When a ‘No’-answer was chosen for a record, it was assessed by a second reviewer and then excluded as ineligible if a ‘No’ answer was chosen again. ASF, African swine fever; ASFV, African swine fever virus; Q, question.

Selection Question	Selection	Ineligible
Q0: Are there no other records with matching title and authors in the total pool of retrieved records?	Yes = proceed to Q1	No = exclude
Q1: Does the record title likely describe a study about ASFV infection in suids and appears relevant to the review objective?	Yes or Not determinable = proceed to Q2	No = exclude
Q2: Is an abstract available for this record AND is it in English, German, Italian or Spanish language?	Yes = proceed to Q3	No = search abstract, may proceed to Q3 or Q5
Q3: Is the record likely peer-reviewed, published, or a doctoral thesis and is NOT a review or editorial-type letter without data?	Yes or Not determinable = proceed to Q4	No = exclude
Q4: Does the record likely describe a study where suids are infected with ASFV? (Population and Outcome)	Yes or Not determinable = proceed to Q5	No = exclude
Q5: Is a full text available for this record before 20 November 2020 AND is it in English, German, Italian or Spanish language?	Yes = proceed to Q6	No = exclude
Q6: From cross-reading through the publication, does the study seem possibly relevant for the review objective?	Yes = proceed to Q7	No = exclude
Q7: Does the record NOT present entirely duplicate data from another original study?	Yes = proceed to Q8	No = exclude
Q8: Is the record peer-reviewed, published, or a doctoral thesis and is NOT a review or editorial-type letter without data?	Yes = proceed to Q9	No = exclude
Q9: Does the record likely describe a study where suids are infected with ASFV? (Population and Outcome)	Yes = proceed to Q10	No = exclude
Q10: Does the record likely describe a study where suids are exposed to possible risk factors? (Exposure)	Yes = proceed to Q11	No = exclude
Q11: Does the record likely describe a study where a comparison group of suids is included or can be deducted from the study design or reference scenario? (Comparator)	Yes = include for extraction attempt	No = exclude

**Table 2:** Description of risk factor categories for African swine fever. Risk categories applicable only to potential risk factors are displayed in italic font; all other categories are applicable to both observation-based and potential ASF risk factors. ASFV, African swine fever virus.

ASF Risk Category	Description
ASFV (virus properties)	Pathogen-related risk factors such as virulence, genotype, strain, dosage, exposure route, tenacity
Biosecurity	Biosecurity-related practices and circumstances such as management of cleaning, disinfection, clothing, food items, pig materials, sick or dead animals, control of feed, water, vehicles, parasites, pests, farm access, fencing, quarantine, and auditing or assessment of biosecurity
Disease control	Disease control measure preparation, implementation, and regulation, or demonstrated compliance, monitoring, and enforcement
Environment	Environmental disease status, such as nearness of ASF cases and factors present or influencing the environment of wild or domestic pigs, including pig density, farm density, human density, presence of vectors or carrier items, land use and coverage (such as forest, farmland, water sites, altitude level, and nearness to pig-related facilities and events such as slaughter units, roads, dumps, parks, agriculture, hunting grounds, and boundaries), as well as climate factors such as precipitation, temperature, humidity, and seasonality-related factors
Husbandry	Factors describing domestic pig husbandry and general farm husbandry characteristics and practices, such as housing type, operation type and size, other species kept, trade type, workers, management of reproduction, slaughter, feeding, records, equipment, and veterinary services
Movement	Factors that relate to potential movement of ASFV in conjunction with animals, any animal materials or products, fomites, farm inputs, persons, trade movements, travel, or vehicle movement
Network	Factors that describe connections between epidemiological units of swine, e.g., trade networks, social networks, farm relations, producer networks, local networks
Pig	Pig-related risk factors such as age, behaviour, breed, sex, habitus of exposure, and lifestyle
Society	Societal factors that may influence ASF spread, such as socio-economic hardship, cultural relevance of swine, population dynamics, education, crisis, standard of living, available services, tourism, wealth, education
Surveillance	Factors influencing the detection of ASF outbreaks, including awareness, surveillance activities and programs, their implementation, testing capacity, testing strategies, veterinary controls, reporting
*Vaccination status*	*Any ASF-related immunomodulatory activities and effects, including ASFV antibody titres, availability of vaccines, testing of vaccine candidates, or activities to induce ASFV immunity*
*Wildlife management*	*Management activities or factors related to wildlife and in particular to wild pigs, such as hunting activity and methods, feeding, fencing, personnel numbers involved, understanding of wildlife biology, habitat disturbance, hunting efficiency and pressure, wildlife dispersion, control, recording and reporting of wildlife status and numbers monitoring, and control of feral and pest swine*

## Data Availability

All relevant data have been made available in the supplementary materials provided with this publication. Desired additional information can be provided upon reasonable request to the authors.

## Supplementary Materials

The following supporting information can be downloaded at: https://www.mdpi.com/article/10.3390/v14102107/s1, Table S1: PRISMA checklist with reporting items for this systematic review. See appended file “prismachecklist.docx”, Table S2: List of studies excluded after full text screening. See appended file “excluded.xlsx”, Table S3: List of studies selected and extracted after full text screening. See appended file “selected.xlsx”, Table S4: Observation-based risk factors (PECO) and risk factor categories for African swine fever. See appended file “obsrf.xlsx”, Table S5: Potential risk factors and risk categories for African swine sever. See appended file “potrf.xlsx”, Table S6: List of keywords identified with the Rapid Automatic Keyword Extraction Algorithm (RAKE) among observation-based and potential risk factors for African swine fever. See appended file “rake.xlsx”.Text S1: Search strings used for querying literature databases to identify publications related to ‘African swine fever’ and ‘Risk’. See appended file “searchstrings.txt”, Text S2: Description of detailed method for literature search strategy, study selection, keyword identification, co-occurrence word networks and risk factor reporting over time. See appended file “method.pdf”.
