# Supplementary material for: Identification of Risk Factors for African Swine Fever: A Systematic Review
Source: Viruses. 2022 Sep 23;14(10):2107. doi: 10.3390/v14102107 (PMC9611626; doi:10.3390/v14102107)
Supplement: Supplementary file 1 [file viruses-14-02107-s001.zip › Text S2-method.pdf]

Supplementary information – Methodology

Bergmann et al., 2022

“Identification of Risk Factors for African Swine Fever by Systematic Review”

Search strategy - Detailed description of search string design

At the outset, keyword topic lists were developed, representing the eligibility components of the PECO review objective: P = pig, E = risk, C = pig, O = ASFV infection. Preliminary keyword lists were compiled through explorative topic database or synonym searches and expert interviews. The preliminary list was then refined through targeted keyword searches of article metadata, such as the Mesh annotation database in PubMed. To ensure that all aspects of the review objective were covered, the keywords were validated by individual keyword-based searches to retrieve previously known relevant records and to also retrieve the largest possible set of results for achieving a wide search.

Where the database search interface allowed, searches were targeted for the keywords to be contained in the title, abstract and keyword fields, as they were found to provide a good balance of relevance and sensitivity. The validation of keywords and string test searches revealed that keywords from the P and C component (pigs and control pigs) imposed limitations on results retrieved through keywords from the E and O components without improving specificity. This was most likely due to the fact that ASF is a swine-specific disease and thus inherently limits the search to pigs, or the P and C component as such. Therefore, P and C keywords were omitted and search strings were designed based on selected keywords from the E and O component alone.

The final set of E component keywords were Chance, Danger, Disease Association, Entry, Epidemiologic Methods, Epidemiologic studies, Epidemiological methods, Epidemiological studies, Epidemiology, Hazard, Incidence, Likelihood, Logistic Model, Logistic regression, Occurrence, Predictability, Prevalence, Probability, Protective Factors, Regression, Risk, Risk aspect, Risk Assessment, Risk calculation, Risk Factor, Risk influence, Risk pathway, Risk valuation, Seroepidemiologic Studies, Seroprevalence, Spread, Threat and Transmission. O component keywords were African swine fever, African swine fever related Viruses, African swine fever virus, Asfarviridae, Asfivirus, ASFV, ASFV infection, ASF-Virus and Iridoviridae.

Search strings were then assembled by forming two separate substrings first, through combining all O keywords and all E keywords as quotation marked character strings through the Boolean operator ‘OR’. The resulting O and E substrings were then combined through the operator ‘AND’ to form the final search string.

The specific search interface requirements of the queried databases imposed further modification of the search string to conduct qualitatively comparable searches. All search strings as applied to each database are presented in Supplementary materials.

Study selection – Detailed description of screening procedure

The review team was selected to include expertise on swine diseases in general, ASF in particular, epidemiology, virology, as well as knowledge of risk factors, risk assessment and literature review.

Study selection was initiated by de-duplicating the library, using a purposive tool of the R software package 'revtools' (Westgate, 2019). Subsequently, de-duplicated record screening followed two practices, consistent with the review objective. First, reviewers applied to each record the stepwise selection questions that were developed to implement the eligibility criteria of study selection. Second, reviewers recorded any mentioned risk factors, to create an exhaustive collection of literature informed potential ASF risk factors.

During sequential, criteria-based study selection, each reviewer first applied selection questions Q1 to Q4 during abstract and title-based screening and then Q5 to Q11 during screening of records selected for full text examination. Two independent reviewers examined each record and recorded the answers to the review questions, as well as comments regarding potential ASF risk factors, on electronic forms. If the first reviewer considered the record to be eligible by answering all of the hierarchical selection questions with 'Yes', it was included for the next screening step. If one selection question was answered with 'No', the record was screened by a second reviewer. If the second reviewer then also answered one selection question with 'No', the record was deemed ineligible and excluded. Otherwise the record was included for the next screening step.

Thus, records were only excluded, if two independent reviewers answered at least one selection question at one of the screening steps with 'No'. Records selected for inclusion based on full text screening were then passed on for a data extraction. This process is graphically summarised in the main figures and tables.

RAKE keyword identification

RAKE is a natural language processing algorithm that enables the identification of essential keywords in a text corpus. RAKE scores were calculated for each identified keyword as a measure of word occurrence relevance. The relative score indicates, how frequently a word occurs and how often it co-occurs with other non-stop words. Scores for keywords consisting of multiple words were derived by the sum of the individual word scores (Wijffels, 2021). RAKE keyword identification and scoring was conducted separately for potential and observation-based ASF risk factor text within each assigned risk category.

Procedure for co-occurrence word networks

For the co-occurrence analysis, tokenised word stems of the risk factor descriptions were used as the underlying text. Annotation of words was based on the Universal Dependencies treebank language model 'English-partut-ud-2.5-191206' (de Marneffe et al., 2021). With each risk factor text, the name of the assigned risk category was included to inform the resulting network about the association of risk factors and risk categories, whereas all other resulting associations in the network were based on detected co-occurrence between risk factors. Self-occurrence of the same word was ignored and only words tagged as nouns, proper nouns, noun definitions, adjectives, verbs and adverbs were considered. Words tagged as non-assignable were also included here to account for ASF-related technical terminology. The network was assembled with the R software package 'gggraph' for the 200 most frequent co-occurrences by using the 'kk' clustering algorithm, with edge weight representing co-occurrence (Pedersen, 2020).

Risk factor reporting over time

Unique risk factor and year combinations were graphed over time for potential and observation-based ASF risk factors, meaning that some risk factors may have been counted multiple times, if they were mentioned repeatedly across different years. To examine potential ASF risks considered in the past, time segments were assigned to break up the covered study period for sequential comparison. A time segment was defined as a period of at least five years between data gaps of more than one year with the maximum number of reported risk factors covered in each segment. For each identified time segment, the proportion of risk factors assigned to each ASF risk category was calculated and presented as a bar graph.
